# Legislative hurdles to using traditional domestic livestock in rewilding programmes in Europe

**DOI:** 10.1007/s13280-022-01822-z

**Published:** 2022-12-29

**Authors:** F. Javier Pérez-Barbería, J. Angel Gómez, Iain J. Gordon

**Affiliations:** 1 Biodiversity Research Institute (University of Oviedo Spanish Research Council Principado de Asturias), 33600 Mieres, Asturias Spain; 2 Department of Agroforestry Science and Technology and Genetics, Institute of Regional Development, Game and Livestock Resources Unit, University of Castilla-La Mancha, IREC, 02071 Albacete, Spain; 3 Consejería Agricultura, Agua y Desarrollo Rural Castilla-La Mancha, Oficina Comarcal Agraria, C/Brunete, 21, La Roda, 02630 Albacete, Spain; 4 Australian National University | ANU Fenner School of Environment & Society, Canberra, ACT Australia; 5 5 Lawson Street, Mysterton, QLD 4812 Australia

**Keywords:** Ecosystem services, Legislation, Restoration, Rewilding-lite, Ruminant, Ungulate

## Abstract

Rewilding is a restoration strategy that aims to return anthropogenic ecosystems to a “self-organized” state, by reinstating trophic complexity through disturbance (e.g. predation, herbivory), dispersal and connectivity. In depopulated areas of Europe, lite versions of rewilding, that maintain but minimize the management of rewilding species (e.g. predators, large herbivores) is gaining support. Livestock rewilding (LR) is a form of rewilding-lite, that uses livestock landraces as keystone species in the restoration of herbivory (the functional integrity of ecosystems) offering ecosystem services, such as ecotourism and the sale of livestock population surpluses, that can mitigate the economic and social effects of rural depopulation. Many challenges remain to implementing LR, including (i) more empirical evidence is required of the feasibility of LR across a variety of habitats and conditions, and (ii) understanding the hurdles that legislation poses for LR, the latter being the aim of this study. To accomplish this, we reviewed the EU legislation on environmental protection, animal health and welfare, identification and traceability, and ownership and civil responsibility, to assess how this might apply to LR. Although there is no specific EU legislation prohibiting LR, the review indicates that it is not clear what legislation applies to LR, as LR’s status lies between that of livestock and wild species. As such the existing legislation can be a serious impediment to the development of LR programmes. We highlight the needs for a legal definition, and status of LR species and their ownership. We propose ways to adapt this legislation to support the application of LR programmes in abandoned areas of EU, for example, by using legal exceptions intended for livestock under extensive animal farming systems.

## Introduction

Rewilding is a restoration strategy that aims to return anthropogenically impacted ecosystems to a “self-organized” state (Svenning et al. 2016). Achieving the purest form of rewilding is constrained by societal norms, for example, reticence of the general public for the re-introduction of native species that might injure people or their economic assets, or the impossibility of establishing the “original” ecosystem trophic assemblage (the biological integrity of the ecosystem) if part of its original fauna is extinct (Perino et al. 2019). An alternative, that is gaining traction in depopulated areas of Europe, is rewilding-lite, which aims to achieve rewilding objectives using large mammalian herbivores under the minimum management (Carver 2014; Pereira and Navarro 2015).

As herbivory is a major driver of bottom-up ecological processes, large mammalian herbivores play a key role in the establishment of ecosystem trophic complexity (Gordon and Prins 2019). Therefore, many rewilding programmes focus on the introduction of large native herbivores (Vlasakker 2014). The issue is that in a number of ecosystems, large native herbivores are missing or extinct, and some of these species could occur in the present conditions in large areas of Europe, if they had survived, (e.g. aurochs, *Bos taurus primigenius*; tarpan, *Equus ferus*; elephants, *Elephas antiquus*; bison, *Bison bonasus*; rhinos, *Dicerorhinus kirchbergensis*) (Bunzel-Drüke 2000). A form of rewilding-lite, that attempts to overcome this problem, is livestock rewilding (LR) (Gordon et al. 2021a, b). LR proposes that traditional breeds of livestock offer the opportunity to reinstate trophic complexity through herbivory (the functional integrity of the ecosystem), dispersal and connectivity, while ecosystem services, such as ecotourism and the sale of livestock products, can contribute to the support of the economy of rural communities in depopulated lands (Tree 2018). LR advocates minimal husbandry, allowing animals free mate choice, the ability to form natural social structures and determine their own spatial movements, in what has been called a “natural” or “self-willed” state (Gordon et al. 2021b). This opens the opportunity to rewild about 11% of agricultural land in Europe (over 20 million ha) that is under high potential risk of abandonment by 2030 (Perpiña Castillo et al. 2018). This is no easy task, as there are a number of challenges that require study, among them are (i) the need for experimental evidence as to the feasibility of LR in a variety of habitats and conditions, (ii) achieving general societal approval of LR and assessing the risks of injuring people and damage to their property, and (iii) understanding the hurdles that actual legislation means for LR (Gordon et al. 2021b). Despite these challenges, and as stated by Soulé (1985), being practical and pragmatism are a must in the conservation of natural resources. A bold approach to restoration programmes is needed urgently, as requiring a thorough understanding of the whole ecological process before acting may come too late to save biodiversity. The aim of this paper is to review the existing legislation that might apply to LR and reflect upon the legislative hurdles that directly or indirectly affect the development of LR in the European Union (EU), how they impact on the implementation of rewilding initiatives, and suggest policy guidelines to facilitate the application of LR programmes. We identify caveats in the legislation that might cause problems for civil responsibility due to the damage caused to persons and private property by the activities of LR species, and finally, we highlight the wider implications for a conscientious and responsible acceptance of LR programmes.

## Materials and methods

**Fig. 1:**
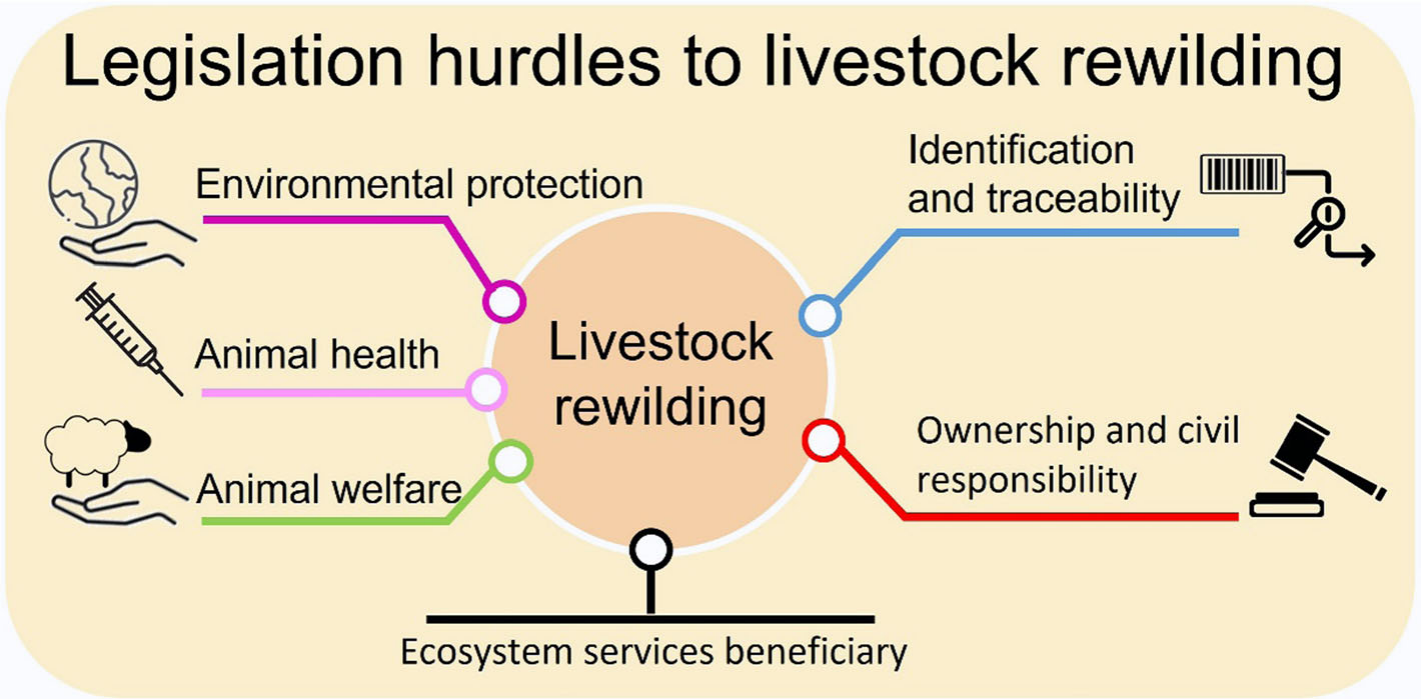
Main bodies of legislation that affect the implementation of livestock rewilding programmes

We reviewed the EU legislation in five areas that we believe are of paramount importance for the development of LR, namely, (i) environmental protection, (ii) animal health, (iii) animal welfare, (iv) identification and traceability, and (v) ownership and civil responsibility (Fig. 1). The pertinent legislation was selected by one of the coauthors (JAG). He is a veterinarian civil servant of the Spanish government, whose responsibility is to audit the application of EU legislation on livestock keeping in intensive and extensive farms. He had access to up-to-date information, and its interpretation, of EU legislation on these matters, and to the adaptations and exceptions of the Spanish legislation. We read through the selected legislation and discussed and interpreted how it applies, or might apply, in the LR context. Although EU legislation is common across its territory, its implementation and enforcement varies between members countries, we have not attempted to carry out a comprehensive review of the particular legislation of each country because it would be quite complex. However, we made special reference to the particularities of the legislation of some countries, because of their interesting context specificity or because of our specific knowledge of the legislation of these countries. We clarify that throughout this paper we used “extensive animal farming systems” as those systems of animal production characterized by low intensity management, that, in general, involve the application of protocols of animal health (e.g. veterinarian treatment), food supplementation when natural resources are scarce, and active control of population numbers by removing animal surpluses.

## What is a rewilded species?

A definition of “livestock rewilded species” is needed to properly understand how they are affected by legislation in Europe. The definition of LR species that we adopt here is the widely accepted historical term of feral animal, that is, a domestic animal, or its descendants, that have escaped or released from a domestic situation to the wild and is living on a self-willed manner. There are caveats in this definition, as Daniels and Corbett (2003) pointed out, introgression can blur the distinction between wild and domesticated forms in future generations, making it difficult to implement conservation measures for these populations. Interestingly, regulation (EU) 2016/429 (2016) defines wild animals as all animals that are not kept by humans, including stray and feral animals, even if they are species that are normally domesticated. Does this really apply to LR animals? It seems that it applies on a case-by-case basis, mainly driven by whether the feral animal has an owner and is or is not on fenced land. For example, in Doñana National Park (southern Spain) free-range cattle and horses are not under the same policy as are their cohabiting wild species, i.e. red deer (*Cervus elaphus*) and fallow deer (*Dama dama*) (Soriguer et al. 2001). However, feral goats from Sierra de Tramuntana (Balearic islands) do seem to receive a similar status to wild species, indeed they are considered to be game animals (López-i-Gelats et al. 2021). In the next section, we highlight which legislation applies to livestock and the impediments that this might create for LR programmes if the LR species is still considered to be livestock.

## Legislation that affects the development of livestock rewilding

There are six main bodies of legislation that affect how domestic species may be used in LR programmes, namely, (i) animal identification and traceability, (ii) animal welfare, (iii) animal health, (iv) environmental protection, (v) ownership and civil responsibility, and (vi) identification of the beneficiaries of LR ecosystem services (Fig. 1, Table 1).

**Table 1:** EU legislation that affects the implementation of livestock rewilding programmes

Regulation	Date	Description
Regulation EU 2016/429	09/03/2016	Standards on transmissible livestock diseases (African swine fever)
Implementing Regulation EU 2018/1882	03/12/2018	Standards for monitoring, prevention, control, spread and eradication of livestock diseases and disease prevention during movements and transport
Delegated Regulation EU 2020/687	17/12/2019	Standards for prevention and control of livestock diseases (amendments to regulation EU 2016/429)
Delegated Regulation EU 2020/688	17/12/2019	Animal health requirements for movements within the EU of terrestrial animals and hatching eggs (amendments to regulation EU 2016/429)
Delegated Regulation EU 2020/689	17/12/2019	Amendments to regulation EU 2016/429
Delegated Regulation EU 2020/692	30/01/2020	Amendments to regulation EU 2016/429 for the entry, movement and handling into the EU of certain animal species, reproductive products and products of animal origin
Implementing Regulation EU 2020/2002	07/12/2020	Development of regulation EU 2016/429 concerning the notification, submission of reports, electronic information system and procedures on transmission of animal diseases
Implementing Regulation EU 605/2021	07/04/2021	Special control measures for African swine fever

### Animal identification and traceability

Animal identification is considered an essential aspect of livestock management (FAO-OIE 2010;1 ; Regulation (UE) 2016/429 (2016)). It underpins the life records of the animal, such as its species, sex, age, medical history, movements off the farm and the identification of the owner or keeper, as the legal person responsible for the care of the animal on a permanent or temporary basis. It also identifies the competent authority under which the control of the animal is regulated, and it is mandatory for international trade and across all EU states (Council Directive 90/425/EEC (1990)). There are some exceptions to the use of individual marking: young lambs, goat kids and piglets, that are going to be immediately slaughtered for human consumption, can be identified by a batch ID (Council Directive 92/102/EEC (1992); Council Regulation 21/2004/EC (2003)) and in cattle calves from extensive systems, marking can be delayed (see below).

For sheep and goats, individual identification must be carried out not later than at 6 months of age or up to a maximum of 9 months in extensive production systems (Spanish Government, RD 685/2013; EU, Council Regulation 21/2004/EC (2003). For these species, the legislation requires the use of a plastic ear tag, placed on the right ear, and in addition, an electronic transponder is placed in the rumen cavity (ID ruminal bolus), and the corresponding animal and owner information must be stored in a database placed on an institutional repository (Spanish Government, RD 728/2007).

The legislation for the identification of bovids (Spanish Government: RD 1980/1998; EU Regulation (EC) 1760 (2000)) establishes the following elements, (i) two ear tags, one on each ear and fitted on the animal at 20 days of age, each tag must be engraved with a unique code that identifies the animal and farm where it was born, (ii) an identification passport document, and (iii) storing the information on an institutional electronic repository. Bovids in extensive production systems are allowed to have ear-tagging delayed  up to 6 months of age of the calf (Regulation (EC) 1760 (2000)). This is permitted for practical reasons, when (i) bovids are reared under extensive farming conditions on open farms, (ii) the natural area in which the animals are kept makes regular handling difficult, and the mother’s protective behaviour of her offspring can be dangerous for the safety of the keepers, and (iii) these conditions do not impede each calf being clearly assigned to its dam.

Horses are a special livestock case in many respects. Equids must be identified, at the latest, at one year of age, or, before leaving the farm of birth, except when this transfer is carried out as a suckling foal. The identification of equids comprises the following elements, a single permanent identification document issued by the pertinent regulatory agency containing a textual and graphic record describing the unique traits of the particular animal; a method of identity verification that ensures an unequivocal link between the identification document and the animal for which it has been issued; and a centralized, institutional database where records are archived. Although the legislation (Commission Regulation 2008/504/EC 2008) states that equids may not be kept unless they are identified in accordance with its provisions, there are exceptions when equids live in wild or semi-wild conditions. For equids to be granted the exception to identification (Directive 92/35/EEC, 1992), first, the competent authority must decide which equid populations are living in wild or semi-wild conditions, based on demonstration that the equids are living under no human control for their survival and reproduction, and that they are effectively separated from domestic equids. Nevertheless, these animals shall be identified by means of an identification document when (i) they are removed from their original populations, excluding transfer under official supervision from one defined population to another, or (ii) they are put to domestic use. When populations of equids are living outside a farm holding but not under wild or semi-wild conditions, as described above, the EU Commission asserts that they should be identified, though exceptions can be provided for when standard animal identification procedures cannot be met.

The identification and registration of pigs is carried out in accordance with the provisions of Council Directive 92/102/EEC, together with any additional identification established in the sanitary programs against certain porcine diseases. This legislation establishes that all pigs must be marked as soon as possible after birth and, in any case, before leaving the farm. Pigs should be marked using an ear tag or a tattoo (animal and farm IDs) according to what the competent authority requires.

Marking wild and feral species is not an easy task, and it can cause injuries to animals and their handlers (Hoel et al. 2013). Under certain conditions, individual marking for identification purposes is possible even for wild populations of considerable size. For example, the Isle of Rum Red Deer Project (Scotland) has been individually ear tagging its deer population for five decades^2^, facilitated by the open landscape of the island but not without complex logistics of deploying and organizing volunteers during calving season. These labour intensive and special habitat conditions are rarely met in many LR programmes, making it difficult, if not impossible, to mark all the population. Fortunately, legislation (see above) recognizes the difficulty of tagging some domestic species living in free-range conditions and establishes exceptions to tagging in the identification in equids, which could also be extended to any LR species to facilitate the initiation and long-term running of rewilding initiatives (Table 2).

**Table 2:** Logistic and risk hurdles to the implementation of livestock rewilding programmes caused by regulations

Regulations	Logistic hurdles	Risk hurdles	Facilitators
*Identification and traceability* Animals must be individually identified wearing marks engraved with a unique code	ID marking requires capture and handling, which is difficult in free roaming animals	Handling animals can cause injuries to them and the handlers Capturing can create an environment of fear and LR animals can flee from those areas, which can be undesirable	LR individual founders can be easily marked before releasing in to the wild LR animals that are going to be transported, dead or alive, from their wild habitat to other areas can be easily marked for identification and traceability purposes LR animals born in the wild should be left unmarked
*Animal welfare* Every animal must have a “life worth living”	Providing animal care to free roaming animals can be difficult and unsustainable Sufficient quality food must be provided to satisfice the animal´s energy requirements	Excess animal care can create undesirable taming and conditions of dependence to humans	To minimize human intervention LR programmes should be developed in habitats that are appropriate to the species (water and food availability, natural shelter) Society should accept that LR animals might suffer similar environmental harsh conditions as those experienced by similar wild species
*Animal health* Control and eradication of contagious diseases between wild, domestic species and people	Generally, it requires animal handling across the animal´s life Health control of the whole population is difficult, expensive and time consuming	Animal handling can cause injuries to them and to the handlers	LR animals should be released in to the wild free of diseases and in optimum body condition to ensure they do not cause risks to the health of wild and domestic species It should be societal acceptable that LR living in the wild might contract diseases as wild species do
*Environmental protection* Assessment of the impact of herbivory in habitats with extensive animal production systems	Lack of detailed protocols to assess herbivory impact in most extensive and semi-extensive animal production systems	Excessive herbivory has a detrimental chain effect on the biodiversity and quality of soils, plants and animals	LR programmes should provide monitoring data to assess their impact on ecosystems
*Ownership and civil responsibility* Rules of tort liability apply when animals cause damages	Who is the owner of LR animals? Who is liable of the damages caused by LR animals of public ownership?	LR animals roam free and can cause damage to people and their property (traffic collisions, damage to crops, attacks to people)	Society should participate in the discussion on implementing LR programmes and it should be clearly informed of their risks
*Ecosystem services beneficiary* Who is entitled of the ecosystem services provided?	Some ecosystem services are diffused (everyone benefits) but others are specific (only few people benefit), beneficiaries should be clearly defined	It can cause tension between societal groups (beneficiaries vs. non-beneficiaries), especially when living in the same area	Society should be clearly informed of the ecosystem services provided by LR programmes and who are the direct beneficiaries

### Animal welfare

Current EU regulation on animal welfare attempts to comply with the principle that every animal must have a “life worth living” (Webster 2016), and legislation can be divided into four main areas, attending to the part of the animal´s life on farm or the use that the animal is intended for (i.e. farm welfare; animal transport; slaughter; and animals use in research). Here, we focus on farm welfare (excluding intensive farming) and slaughter (Table 2).

Legislation establishes that when it is necessary and possible, livestock kept outdoors shall be protected against harsh weather, predators and the risk of disease (Council Directive 98/58/EC, 1998). The same Directive states that enough quality food and water should be provided to maintain the animal´s good health and to satisfy their nutritional needs, though the term “satisfy” is not clearly defined. The Directive also establishes the provisioning of a minimum quantity of dietary components (minerals and fibre) during the first 20 weeks of life of a bovid offspring and colostrum within 6 h after birth (Council Directive 91/629/EEC, 1991).

Applying this Directive to LR could be a serious hindrance for the foundation of sustainable rewilding programmes. Although consideration of a species energy requirement, habitat use and food availability year-round can ensure the wellbeing of LR animals in most conditions, episodes of food shortage, associated with harsh weather or droughts, are inevitable. Society should be aware of the possibility of these events and would have to accept the perils to what livestock might be exposed to in LR situations, which should not be greater than those that wild species bear in similar natural conditions. Pérez-Barbería and Gordon (in press) found contrasting opinions, across societal groups, as to whether human intervention should ensure the avoidance of mortality caused by natural stochastic effects on LR populations. For example, the percentage of acceptance of not providing food during periods of scarcity ranged across societal groups between 39% and 75%—male nature enthusiasts were the most likely to accept that LR lived in a self-willingness state, and the female hunters and female farmers were less keen to accept this situation. Having said that, it is essential to avoid the situation that occurred in the natural reserve of Oostvaardersplassen in the Netherlands, for example, where the growth of herbivore populations (although not LR) outstripped bottom-up resources, causing mass die offs and related ecosystem impacts, and led to a social outcry (Theunissen 2019). For LR this scenario would risk the social license to continue operating with minimal human intervention. Supplementary feeding is an immediate solution in events of food shortage, but with devastating effects on ecosystem function and animal behaviour (i.e. taming). A plausible solution is to manage the population by reactive culling, that is, removing individuals that show clear signs of starvation. This practice could both ensure that no animals are dying of starvation and keep the population wild and free from supplementary feeding.

As recommended by the legislation for extensive production systems, the provisioning of shelter or handling buildings in LR programmes should be considered on case-by-case basis. In new rewilding programmes it may be necessary to provide artificial shelter at the location where the animals are released, until they have developed their own roaming preferences and habitat use (so-called soft release). If, at this stage, the animals do not use the artificial shelter and these facilities are obsolete for other handling purposes, they could be removed. The use of portable buildings would minimize their visual impact, labour and expense. If buildings are installed, then legislation requires that they are disinfected and pest control is conducted on regular basis (Council Directive 98/58/EC, 1998); this is a nuisance and expense but hardly something that affects ecosystem function, unless it prevents the natural roaming behaviour of the animals, but this is something that applies to natural shelters as well.

The erection of fences to facilitate the movement of LR animals to handling facilities, or, using a means of transport to move animals off the rewilding area, can be useful, but serious thought should be given to ensure that they are effective and not a danger to other wildlife in the area (Hanophy 2014). In large areas it is unlikely that relatively short lengths of fencing will facilitate the driving of animals to handling facilities, unless they can be lured to a restricted area, for example, by providing food during periods of shortage (Smith 2001). Rewilding programmes must assess the adequacy of the habitat for the rewilded species, minimizing the need for artificial shelters against cold or hot conditions, at least after the adaptation phase. All this should be considered before providing expensive facilities that might be of limited use.

The provisioning of protection from predators, as indicated by the Council Directive 98/58/EC, is a point that clearly challenges the purposes of LR and that might require social license to operate. If the selection of the LR species have been properly conducted for a specific rewilding habitat we should allow predation to operate on the dynamics of these populations as this will facilitate trophic complexity and restore other ecological functions (a key goal of rewilding).

The regulatory provisions on the animal welfare of equids differ between individual EU Member States—only a few have adopted specific legislation. This is mainly because of the variety of uses equids are intended for, that ranges from companionship, sport, tourism, therapy, draft or meat production^3^. The World Organisation for Animal Health (OIE) establishes a code of practice to ensure the animal welfare of working equids (OIE chapter 7.12 of the OIE Terrestrial Animal Health Code), although, in the EU, the number of working equids is small and in sharp decline (Haddy et al. 2020). Most recommendations are related to the appropriate handling and provision of fair working conditions, which are not relevant for LR equids, however, recommendations on providing shelter from heat and cold stress, and protection from predators and injury, might apply to LR equids.

One of the main ethical issues in animal production systems is animal welfare during the process of slaughter (Browning and Veit 2020), even to the extent that may be possible to eliminate the killing of animals for human consumption by means of alternative meat production processes, such as cell-based meat (Heidemann et al. 2020). Some LR programmes consider the possibility of using meat from the surplus animals in the populations (Tree, 2018), which necessarily involves slaughter. There is a strict EU legislation on the slaughter of animals for human consumption or as a humane method to eliminate suffering (Council Regulation 2009/1099/EC (2009)) (Fenwick et al. 2009). The ethics of animal suffering, when being slaughtered, also includes the suffering involved during transportation to slaughterhouses. Legislation and societal attitudes towards the use of meat from the surplus animals of LR populations should reflect on what slaughter methods will minimize animal suffering, and if they are compatible with legislation on meat for human consumption. Gathering free-range animals (for their capture and consumption) that are not used to regular handling is not an easy task, and it can create harmful situations for them and their handlers. Stalking hunting (shooting from a distance by an experience marksman with a high power rifle) can be a humane method of slaughter in LR programmes, but it is not without its own problems: (i) very large carcasses, e.g. cattle or horses, can be very difficult to transport through rough terrain (though very large game have been removed efficiently from their populations by hunting activity for millennia (Lupo 2006)), (ii) butchering the animal on site and moving the cuts to make transport feasible might not meet health and quality control legislation on meat for human consumption (Merwe et al. 2011), (iii) the disposal of animal by-products (entrails) on site might not be allowed by local legislation (RD 50/2018), and (iv) shooting several animals at one place and time can be difficult if not impossible. It might be the case that the surplus from LR populations is not intended or appropriate for human consumption, which could involve inconvenient and expensive logistics to transport carcasses, and create a high carbon footprint for their legal disposal. There is existing EU legislation that promotes the conservation of scavenging bird populations by means of disposing of carcasses of sheep and goats from extensive farming systems in the area, though only in certain areas (Table 3), which has also undoubted benefits for local biodiversity through trophic chain cascading effects (Stiegler et al. 2020). This legislation could be extended to any carcass from LR, including those of bovids and equids.

**Table 3:** EU and Spanish regulations on supplementary feed to scavenging bird populations and its beneficial implications to LR, environment and biodiversity

Regulation	Concept	Implications to LR, environment and biodiversity
Council Directive 79/409/EEC, 02/04/1979 Spanish Law 4/1989, 27/03/1989	Implementing habitat measures to guarantee the conservation of species within the National Catalogues of Threatened Species	
Commission Decision 2003/322/EC, 13/05/2003 Commission Decision 2005/830/EC, 26/11/2005 Regulation EC 1069/2009 of the European Parliament and of the Council,21/10/2009	Greece, Spain, France, Italy and Portugal are authorized to set up feeding points for scavenging birds in which sheep and goat carcasses can be deposited, upon condition that 4% of livestock exploitations in those areas were found to be free of transmissible spongiform encephalopathies (TSE). It is expected to promote biodiversity	Promoting conservation of endangered species and opportunity to facilitate control of LR populations
Directive 2009/147/EC of the European Parliament and of the Council, 30–11-2009	Complementary feeding for scavenging birds should not affect their behavioural trophic patterns	
Commission regulation (EU) 142/2011, 25/02/2011 Regulation EC 1069/2009 of the European Parliament and of the Council,21/10/2009	It develops the sanitary conditions and measures for the implementation of supplementary feed for scavenging birds in the open field, including the use of carcasses from extensive farming exploitations in these areas	Promoting biodiversity and the sustainability of traditional pastoralism, carbon footprint reduction in animal subproducts
Spanish Royal Decree 1632/2011, 14/11/2011	It regulates sanitary conditions, levels and animal subproducts of supplementary feeding, definition of the populations of scavengers of conservation interest and the demarcation of their breeding grounds and movements, recording animal farms under extensive exploitation systems and their expected carcasses contribution as supplementary feeding for scavengers and monitoring TSE	Ecosystem services facilitation

### Animal health

Animal health aims to eradicate or control diseases that affect animal populations, or that can be transmitted to humans, negatively impacting public health or economic interests (Rhyan and Spraker 2010). The existence of disease reservoirs in the natural environment, and the increasing interaction between wildlife and humans or domestic animals, leads to disease emergence and requires strategies for disease surveillance and management in wildlife, which make sanitary actions in livestock and wild species inseparable (Rhyan and Spraker 2010). Therefore, animal health legislation applies to both livestock and some wild species, although with logistical limitations in the latter, except for when wild species are in intensive or semi-intensive production systems, dedicated to consumption, reintroductions or release in hunting estates; in this case the same legislations apply as to livestock (Table 2). Animal health programmes affect the natural course of the population dynamics of LR and involved direct human intervention, which contrasts with the purist version of rewilding, but because animal transmitted diseases pose such a serious risk to human health and economic interests animal health legislation and their implications for LR deserves consideration.

Animal health legislation can be divided into two sections (i) that which affects the hygiene of livestock facilities (disinfection and elimination of pests), and (ii) animal sanitation campaigns, we focus on the latter. For species suitable for LR programmes, EU legislation establishes monitoring and disease eradication programs for tuberculosis and brucellosis, bovine leukosis, bovine infectious rhinotracheitis and infectious pustular vulvovaginitis, bovine viral diarrohea and bluetongue, as well as eradication programmes for infection with rabies virus in wild animals of Bovidae, Suidae and Equidae families (Commission Delegated Regulation 2020/689, 2020). There are specific programs in the EU and The World Organization for Animal Health (OIE) that apply to wild, game species, which can be relevant and extended to the health of LR populations (e.g. Aujeszky's disease, classical swine fever, African swine fever, trichinosis, and vesicular disease, for wild boar and feral pigs; brucellosis for wild boar, cervids and bovids; tuberculosis for all species except bovids, and scabies in bovids). For example, Spain has developed specific control programs for these diseases (RD 2611/1996), which involves zoning the country based on the prevalence levels and disease impacts, including monitoring samples from game animals, and reducing the density of game populations in areas of high risk (e.g. Action Plan on Tuberculosis in Wild Species). As supplementation to this Regulation, The Commission Delegated Regulation (EU) 2020/687 establishes specific disease control measures for wild animals of listed species in the case of the occurrence of a category A disease (i.e. diseases that do not normally occur in the EU). Namely, (i) conduct post-mortem examinations of wild animals of listed species shot dead or found dead, (ii) ensure that the entire bodies of the dead wild animal, or parts thereof, are disposed of in accordance with Regulation (EC) 1069/2009 (2009), and (iii) in the event of an outbreak, prohibiting the movements of wild animals of listed species and their products, regulate movements of kept animals of listed species, regulate hunting activities and other outdoors activities, restrict feeding wild animals of listed species and implementing an eradication plan.

Though many of these measures are desirable for human wellbeing and economic interests, in practice they are not easy to apply. It is obvious that in any LR programme, animals that are to be released into the wild should be free of diseases and in optimum body condition, to ensure they do not cause risks to the health of wild and domestic species, and that they are in good condition to ensure acclimatization to the new conditions. It should be acknowledged by society that LR animals living in the wild might contract the same diseases that their wild relatives are exposed to, as this is to some extent inevitable (Table 2). The public should be also be aware of the inconspicuous negative effects that livestock health treatments have on the environment, as for example, some chemical compounds, administered to livestock for parasite control, can have lethal and sublethal effects on coprophagous insects, thereby impairing ecosystem functions that underpin even agricultural production (Manning et al. 2018). Trying to impose on LR populations the same legislation on health that applies to livestock will, undoubtable, be a hurdle to the initiation of LR programmes and will make them unsustainable. The key question that requires reflection by experts is whether releasing into the wild LR animals increases the risk to the health of other species and people, in a manner that overwhelms the benefits that LR ecosystem services provide the society.

### Environmental protection

The role that large herbivores have in the conservation of ecosystems has long been recognized (Gordon and Duncan 1988; Gordon and Prins 2019). There is evidence that medium levels of herbivory contribute to the promotion of biodiversity. This is because grazing and browsing offtake reduces the dominance of certain plant species, diminishes the accumulation of plant litter, faecal input provides habitat niche for dung feeding arthropods and creates mosaics in the distribution of soil nutrients and seeds, and trampling creates spatial heterogeneity in soil and plant communities (Cumming and Cumming 2003). All these translate into an increase in the heterogeneity of the physical–chemical properties of the soils, with a consequential increase in the number of micro-habitats, which provides opportunities for the establishment of different plant species and its consequences for higher taxonomic levels though the trophic chain (Vavra et al. 2007; Bakker and Svenning 2018). On the other hand, high densities of herbivores have a negative impact on soils, changing their chemical composition and reducing infiltration, with increased runoff and erosion that contributes to desertification (Weber and Horst 2011). Excessive herbivory also produces changes in vegetation structure, reductions of the taxonomic diversity of plants, and limits or completely impedes forest regeneration (Wieren and Bakker 2008). Because of this, the use of large ungulates, at medium stocking densities, has been promoted as an effective tool in habitat restoration programmes (Reimoser and Putman 2011). On the other hand, it has been suggested that stocking densities are highly context-dependent, as a consequence it has been recommended that rewilding of large herbivores occurs with near-natural grazing pressures without predefined density targets (Fløjgaard et al. 2022). EU legislation on the impact of large herbivores on habitats is unclear—some EU member states have legislation on habitat protection that might apply in specific cases of detrimental grazing pressure in LR programmes (Table 2). For example, in Spain the legislation on extensive pig farms establishes that management must guarantee a rational use of the entire physical environment of the farm, taking advantage of the natural resources and establishing rotational management systems to avoid ecosystem degradation. Namely, on an annual basis, each farm must establish their maximum stocking densities in relation to the natural resources available, in any case, stocking density must not exceed 15 fattening pigs/ha (2.4 livestock unit/ha), or its equivalent (RD 1221/2009). Although this could be used as starting point, these guidelines are too specific to be applied to non-suid ungulates and across a variety of biotic and abiotic conditions and habitats. Fortunately, LR programmes are designed to promote biodiversity within a specific area and across a variety of changing environmental conditions, which requires, among other things, monitoring the grazing effect by large herbivores on these habitats. Consequently, LR programmes can provide useful information to establish adaptive management protocols that prevented habitat deterioration by excessive herbivory.

In Europe, the cornerstone of nature conservation policy is the Council Directive 92/43/EEC of 21 May 1992, that together with the Birds Directive constitute the legislative pillars of the Natura 2000 network of protected areas. This Directive aims for the conservation of natural habitats, wild fauna and flora to promote the maintenance of biodiversity, at the same time that takes into account economic, social and cultural factors. The Directive does not mention livestock rewilding, but there is a complementary document that helps with the interpretation of the Directive (“EC guidance on species protection”)^4^. This document specifically interprets that domestic species or wild species that deliberately or accidentally have been introduced by man, into places where they have never been naturally present or are unlikely to spread there in the near future, are to be considered outside their natural range and thus excluded from the scope of the Directive. It is clear, therefore, that livestock species in rewilding programmes are not protected under the umbrella of the above-mentioned legislation. The question now is whether livestock rewilding is compatible, or even desirable, within the Directive, which boils down to assessing the risks and benefits that livestock rewilding have on local wildlife. We, therefore, face the following dialectic: should we consider the composition and structure of the local wildlife as the one to be preserved, or, should we consider that a particular livestock rewilding programme will enrich the ecosystem to such an extent that the potential reduction of the populations of some wild species is justified? This is not an easy question to answer without first having an ecologically based social agreement on what local fauna composition the rewilding programme is intended to achieve.

The Annex IV (a) of the Directive covers a wide variety of species and very different distribution areas, which involves the implementation of the Directive on a case-by-case basis. Therefore, the implementation of a livestock rewilding programme should evaluate the pros and cons and compatibilities between introduced and protected species. In the case of ongoing land use practices, such as agriculture or forestry, the challenge is to implement species protection provisions in a way that minimizes future conflict.

The key, in our view, is the use of management tools and information that meet conservation needs while addressing economic, social and cultural requirements. These tools must be accompanied by a legal framework that ensures adequate enforcement, by regulatory authorities, in the case of non-compliance, and we believe that legislation should address at least the general guidelines for using livestock rewilding as a potential tool for nature conservation.

### Ownership and civil responsibility, and ecosystem services beneficiary

Although LR programmes are intended to be developed on areas with little current agricultural activity, damage to neighbouring crops and forestry by LR animals is a distinct possibility. In these cases, and to increase people's tolerance for rewilding, some preventative measures should be implemented, such as removal of problematic animals, and compensation for damage caused by LR animals.

In terms of prevention, a sensible action is to develop species habitat preference and distribution models to identify the areas with the highest likelihood for the presence of a species (Pérez-Barbería et al. 2013), and the potential collateral damage to crops and property. The erection of fences to restrict access to crops is expensive, requires maintenance, especially in areas with wild boar (*Sus scrofa*) or feral swine (Lavelle et al. 2011), and it can have negative impacts on some wild species and their spatial movements (Hanophy 2014). However, temporary fencing can be advisable in LR programmes that also involve reforestation.

The law of civil responsibility reflects a commitment to those or their property, which have been harmed because of the activity of animal species that are under special protection (Table 2). The law embodies a social determination that the affected people should be fairly compensated. Damage caused by non-protected species are not covered by public compensation schemes but by the owner or keeper, to whom general rules of tort liability apply. For compensation schemes to be sustainable, they should be applied only when damage cannot be avoided through the use of preventive measures (Klemm 1996). The law clearly establishes that the damage must be caused by species under special protection, which is at the discretion of EU member states^5^. Though compensation schemes should be adapted to the local circumstances, some useful notes for LR programmes can be drawn from the experience of the compensation schemes applied to the introduction of large carnivores in Europe (Fourli 1999). Establishing the legally responsible entity for the compensation is mandatory and should be the first step for a compensation scheme to be effective. Therefore, there is a need to define who is the owner of rewilded species used in LR programmes, especially when these programmes can be carried out in large open areas that comprise several administrations and LR species can have large home ranges and unpredictable long-distance movements. In many countries, wild animals are *res nullius* (i.e. ownerless property), which means no one can be held legally liable for the damage they might cause. In the scenario that LR species would be considered under the same legislation that affects wild species, and they are legally defined as public property, then, public administrations will not be liable for a tort of negligence, nuisance or trespass, for damages caused by these species. In this scenario, the individual should assume the costs of the damage, and this could cause conflicts between societal groups, especially those that live in LR areas, though it is equally true that they could or should benefit the most from the ecosystem services provided by LR programmes^6^. Whether the compensation costs fall on the shoulders of the people that live in that territory or are shared by the entire society should be decided on the basis of which party benefits most from the ecosystem services provided by a LR programme (Fourli 1999). When species are considered protected taxa, they become *res omnium* (i.e. common heritage), and self-defense measures are not applicable anymore, and the State may be considered to be liable for the adverse consequences of its own legislation. Some useful examples from France, Greece, Spain and Austria are as follows: according to the Nature Protection Act of 1976 the French State is not liable for the damage caused by protected animals (Klemm 1996). Nevertheless, the State has made compensation arrangements for damage caused by bears, wolves and lynx. The French Departmental Directorate of Agriculture and Forestry (DDAF), and all bodies making compensation payments on behalf of it are funded by the Ministry of Environment, which means that even if legally the French State is not liable for the damage, it actually takes care of the matter. In Greece, the body responsible for decisions related to the compensation of damage by all kinds of natural causes (e.g. weather, wild animals, diseases) is the Greek Agricultural Insurance Organisation (ELGA). This is a semi-public body whose financial sources come mainly from the obligatory insurance premia of Greek farmers, livestock raisers, hunting license taxes and regional funds (Klemm 1996). In Spain, the regional administrations of the autonomous communities are responsible for the damage caused by wild animals, within the boundaries of these administrations (i.e. Natural Parks and Game Reserves), and in some occasions covered by LIFE projects, while in Austria, hunters associations and insurance companies are the responsible bodies for compensation (Fourli 1999).

One of the most concerning risks caused by large herbivores to human health is traffic collisions. Langbein et al. (2011) reviewed the impact of traffic collisions in EU on people’s health and on ungulate populations themselves. They estimate about 1 M collisions per year and between 1 and 5% of these reported road traffic accidents caused human injuries, note that the number of human fatalities is considerably lower, but it is still distressing. On the economic side, estimates revealed an average cost of repairs of €2000–2500 per collision (Bissonette et al. 2008; Langbein et al. 2011). The role of non-governmental structures involved in compensation mechanisms becomes an additional and important tool to placate the public's concern about the damage caused by wildlife to human´s health and property. Furthermore, establishing an insurance linked with a LR programme could be part of the solution.

Great care should be taken for compensation to be used as a means for ensuring the peaceful coexistence between humans and large herbivores, and not merely as another tool for providing agricultural subsidies to marginal communities (Cozza et al. 1996). For example, in some regions compensation is much higher than the cost of damage caused to crops and more lenient from a strict verification of the damages point of view (e.g. Navarra, Aragón and Cataluña in Spain or in the French Pyrenees) (Fourli 1999), which might promote opportunistic practices of the plaintiffs. Compensation schemes for the damage caused to property by LR animals should be based on actual market values of the goods and the associated nuisance of the damage rather than on fixed rate basis. Finally, society should be clearly informed of the costs and benefits of LR programmes, this could minimize public aversion when damages took place.

## Conclusions

The legislation that affects LR programmes in the EU is that related to animal health and wellbeing, damage caused to human health, economic interests and to ecosystems, and the economic exploitation of the surplus of their populations. From a rewilding and practical perspective, we advocate for applying to LR animals the existing legislation on the management of wild species, as close as is possible. This makes sense if the main objective of LR is to replace the functional role of missing or extinct wild species, either temporarily or permanently, and to exploit, in a sustainable way, the ecosystems services LR provides. Many pieces of existing legislation, that apply to livestock, impose significant hurdles to rewilding aims, especially individual identification and health control. The clear definition as to what a LR species is, and its ownership, are the main issues that compromise the efficient application of existing legislation to LR species. This is especially the case as related to tort liability on animals that could be considered ownerless property, but which might be providing direct benefits to particular sectors of society. Key attributes for the successful implementation of rewilding programmes is achieving wide support from the public by (i) information campaigns on the benefits, costs and risks that LR involves to individuals, their property and society, and (ii) encouraging society to be an active participant in the implementation of LR, especially those societal groups that live in areas where these programmes could be developed and where conflicts of interest might be at stake.

## Footnotes

1 https://ec.europa.eu/food/animals/identification_es

2 (https://rumdeer.bio.ed.ac.uk/)

3 (https://ec.europa.eu/food/animals/animal-welfare/eu-platform-animal-welfare_en)

4 (https://ec.europa.eu/environment/nature/conservation/species/guidance/index_en.htm)

5 (https://ec.europa.eu/environment/nature/conservation/index_en.htm)

6 (https://www.lawreform.ie/_fileupload/consultation%20papers/wpAnimals.htm)
